# An Xp11.2 translocation renal cell carcinoma with *SMARCB1 (INI1)* inactivation in adult end-stage renal disease: a case report

**DOI:** 10.1186/s13000-016-0551-x

**Published:** 2016-10-12

**Authors:** Lu Yu, Jun Li, Sanpeng Xu, Mariajose Navia Miranda, Guoping Wang, Yaqi Duan

**Affiliations:** 1 Institute of Pathology, Tongji Hospital, Tongji Medical College, Huazhong University of Science and Technology, Wuhan, 430030 People’s Republic of China; 2 Department of Pathology, School of Basic Medical Science, Huazhong University of Science and Technology, Wuhan, 430030 People’s Republic of China

**Keywords:** Renal cell carcinoma, Kidney, TFE3, Xp11.2, *SMARCB1*

## Abstract

**Background:**

Xp11.2 translocation/transcription factor E3 (TFE3) rearrangement renal cell carcinoma (RCC) is a rare subtype of RCC with limited clinical and pathological data.

**Case presentation:**

Here we present an unusual high-grade Xp11.2 translocation RCC with a rhabdoid feature and *SMARCB1 (INI1)* inactivation in a 40-year-old man with end-stage kidney disease. The histological examination of the dissected left renal tumor showed an organoid architecture of the eosinophilic or clear neoplastic cells with necrosis and high mitotic activity. In some areas, non-adhesive tumor cells with eccentric nuclei were observed. Immunohistochemically (IHC), the tumor cells are positive for TFE3 and the renal tubular markers (PAX2 and PAX8), and completely negative for SMARCB1, an oncosuppressor protein. Break-apart florescence in situ hybridization and reverse transcription polymerase chain reaction confirmed TFE3 rearrangement on Xp11.2 and the presence of ASPSCR1-TFE3 fusion gene. DNA sequencing revealed a frameshift mutation in exon 4 of *SMARCB1* gene*.*

**Conclusion:**

It is important to recognize this rare RCC with both TFE3 rearrangement and *SMARCB1* inactivation, as the prognosis and therapeutic strategies, particularly targeted therapies for such tumors, might be different.

## Background

Renal cell carcinomas (RCCs) represent 90 % of all malignancies of the kidney in adults and <5 % of the malignancies in children. WHO subdivision of this entity includes a group of neoplasms distinguished by chromosomal Xp11.2 translocations resulting in the fusion of the transcription factor E3 (TFE3) gene to one of the different partners, including ASPSCR1, PRCC, NonO (p54^nrb^), PSF and CLTC [1], with the consecutive overexpression of the chimeric protein TFE3. The diagnosis of Xp11.2 translocation RCC is not defined based on morphological features, but rather the genetic identification of Xp11.2 translocation using florescent in situ hybridization (FISH) [1].

The gene *hSNF/INI1/SMARCB1/BAF47* is a putative tumor suppressor gene expressed in all normal cells. The inactivation of *SMARCB1* has been observed in malignant rhabdoid tumors (MRT), childhood atypical teratoid/rhabdoid tumor (AT/RT) of the central nervous system (CNS), epithelioid sarcoma [2], subsets of collecting duct carcinoma [3] and epithelioid malignant peripheral nerve sheath tumor (MPNST) [4], renal medullary carcinoma [5] and undifferentiated pediatric sarcomas [6], etc. The loss of SMARCB1 nuclear expression is of diagnostic value for renal or extra-renal MRT and AT/RT [7].

Here we presented a high-grade malignant renal cell cancer with TFE3 translocation and *SMARCB1* inactivation in an end-stage kidney.

## Case presentation

**Fig. 1:**
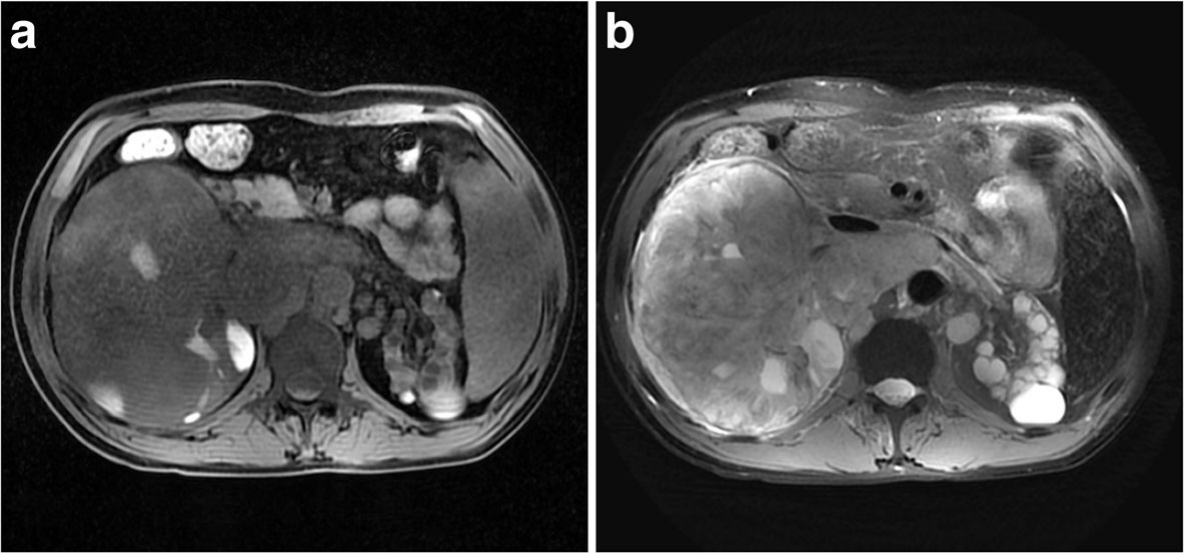
Magnetic Resonance Image (MRI) of the abdomen in a 40-year-old man with Xp11.2 translocation RCC. **a**, **b**, Axial T2WI (**a**) and plain T1WI (**b**) showed a large, well-defined, irregular mass (T2, high-low heterogeneous signal intensity; T1, iso-signal intensity) with patchy hemorrhage and necrosis in the mass and enlargement of abdominal lymph nodes

A 40-year-old man with chronic renal failure and undergoing long-term hemodialysis (7 years) was admitted to the hospital as a result of chronic flank pain. Subsequent Magnetic Resonance Image (MRI) of the abdomen revealed an approximately 12 cm × 6 cm × 5 cm solid mass in the right kidney with calcification, a cystic lesion in the left kidney and enlargement of the retroperitoneal lymph nodes (Fig. 1). Radical nephrectomy of the right kidney was performed.

Grossly, the renal parenchyma was almost replaced with a grayish tan and fleshy tumor with focal necrosis, hemorrhage and calcification. The tumor invaded the renal pelvis, calyces and the hilar area of the kidney and extended to the capsule.

**Fig. 2:**
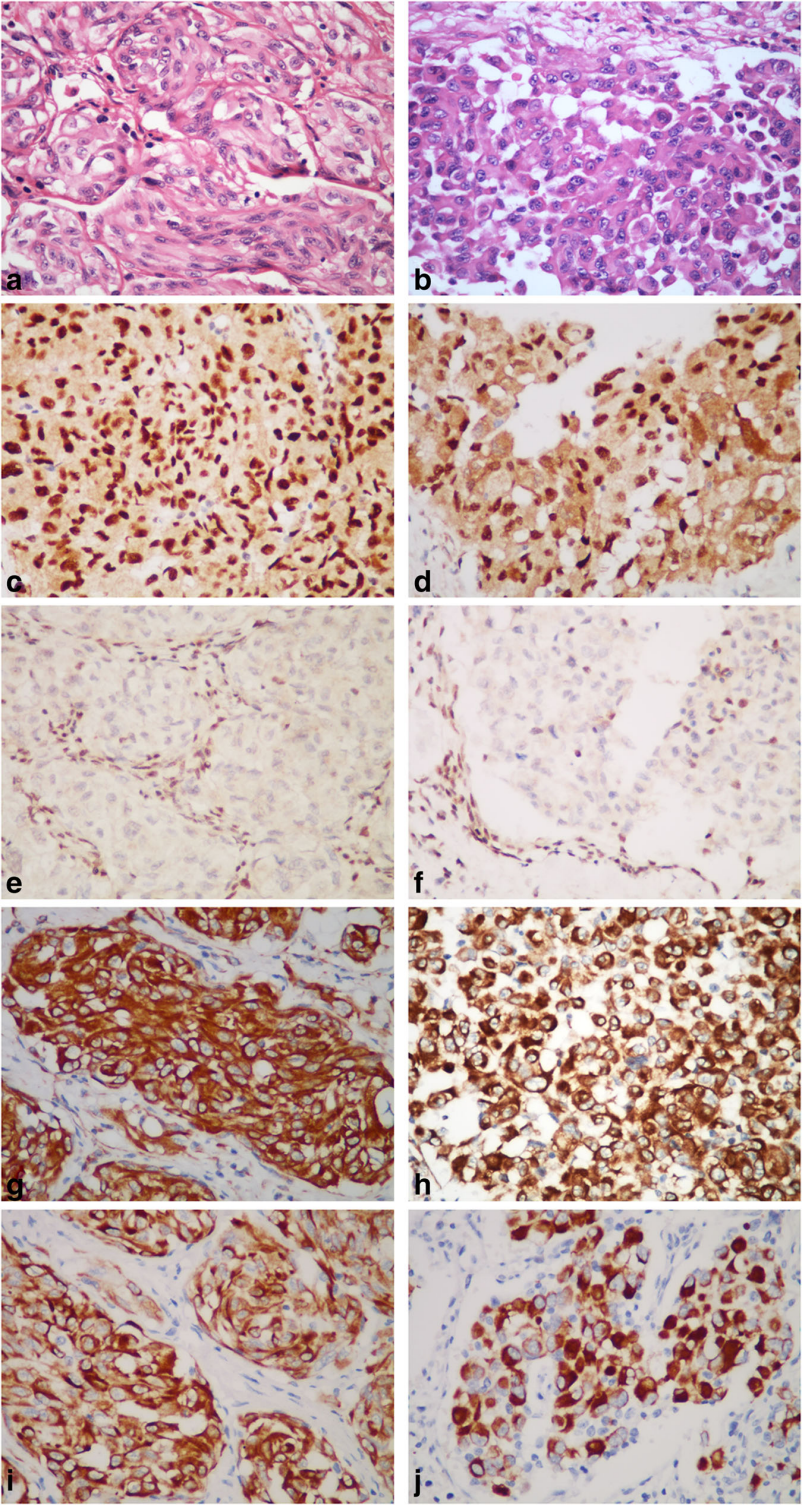
Histopathological and immunohistochemical features of the renal tumor. **a**, **b** Nested eosinophilic tumor cells (**a**) and non-cohesive tumor cells (**b**) with abundant pink cytoplasm and eosinophilic intracytoplasmic inclusions (H&E staining, 400X magnification). **c**, **d**, **e**, **f** Neoplastic cells in both organoid and non-adhesive areas demonstrated strong nuclear staining of TFE3 (**c**, **d**) and negative staining of INI1 (**e**, **f**) (400X magnification). **g**, **h**, **i**, **j** All tumor cells showed strong positive staining for vimentin (**g**, **h**) and pan-CK (**i**, **j**) with prominent perinuclear and cytoplasmic staining in non-adhesive area (400X magnification)

The tissues were fixed in 10 % buffered formalin solution, embedded in paraffin block, 4 μm thick sections were obtained and subsequently stained with hematoxylin-eosin. Under the microscope the tumor cells were arranged in an organoid pattern with a well-defined cell border and eosinophilic voluminous cytoplasm. The nucleus was enlarged and possessed vesicular chromatin with apparent nucleoli, indicating a high nuclear grade. In some areas, rhabdoid cells were observed. These rhabdoid cells were non-adhesive and showed eccentric nuclei and intracytoplasmic inclusions of eosinophilic hyaline globules (Fig. 2a, b). The high malignancy was indicated by high mitotic activities (Ki 67 index approximately 40 %) and increased necrosis in the lesion. The tumor invaded to the adipose tissue of the renal hilum.

Next, immunohistochemistry was performed on the sections to assess tumor classification using an avidin-biotin peroxidase technique with hematoxylin counterstaining. The antibodies used in the present study included anti-vimentin (V9, 1:400, Dako), anti-pan-CK (AE1/AE3, 1:200, DAKO), anti-PAX2 (EP3251, 1:1000, Abcam), anti-PAX8 (ZR-1, 1:100, Abcam), anti-HMB45 (HMB45 + 50, 1:100, DAKO), anti-MelanA (A103, 1:100, DAKO), anti-MyoD1 (58A, 1:100, Santa Cruz), anti-Myogenin (F5D, 1:50, Santa Cruz), anti-TFE3 (H-300, 1:100, Santa Cruz), and anti-SMARCB1 (H-300, 1:100, Santa Cruz). As demonstrated in Fig. 2g-j, vimentin and pan-CK were both positive in all tumor cells, with more prominent perinuclear and intense cytoplasmic staining in rhabdoid cells (Fig. 2h, j). The neoplastic cells showed positive staining for PAX2 and PAX8, indicating renal tubular differentiation, and negative staining for CD10, HMB45, MelanA, myogenin, myoD1, desmin, CD31 and CD34. Interestingly, we observed that all tumor cells showed the complete loss of nuclear staining for SMARCB1 in both the organoid and non-adhesive areas with rhabdoid cells compared with the ubiquitous expression of SMARCB1 in surrounding normal tissue (Fig. 2e, f), indicating a renal malignant rhabdoid tumor. Surprisingly, the tumor cells demonstrated strong nuclear and weak cytoplasmic staining for TFE3 (Fig. 2c, d), a relatively specific IHC marker for Xp11.2 translocation RCC.

**Fig. 3:**
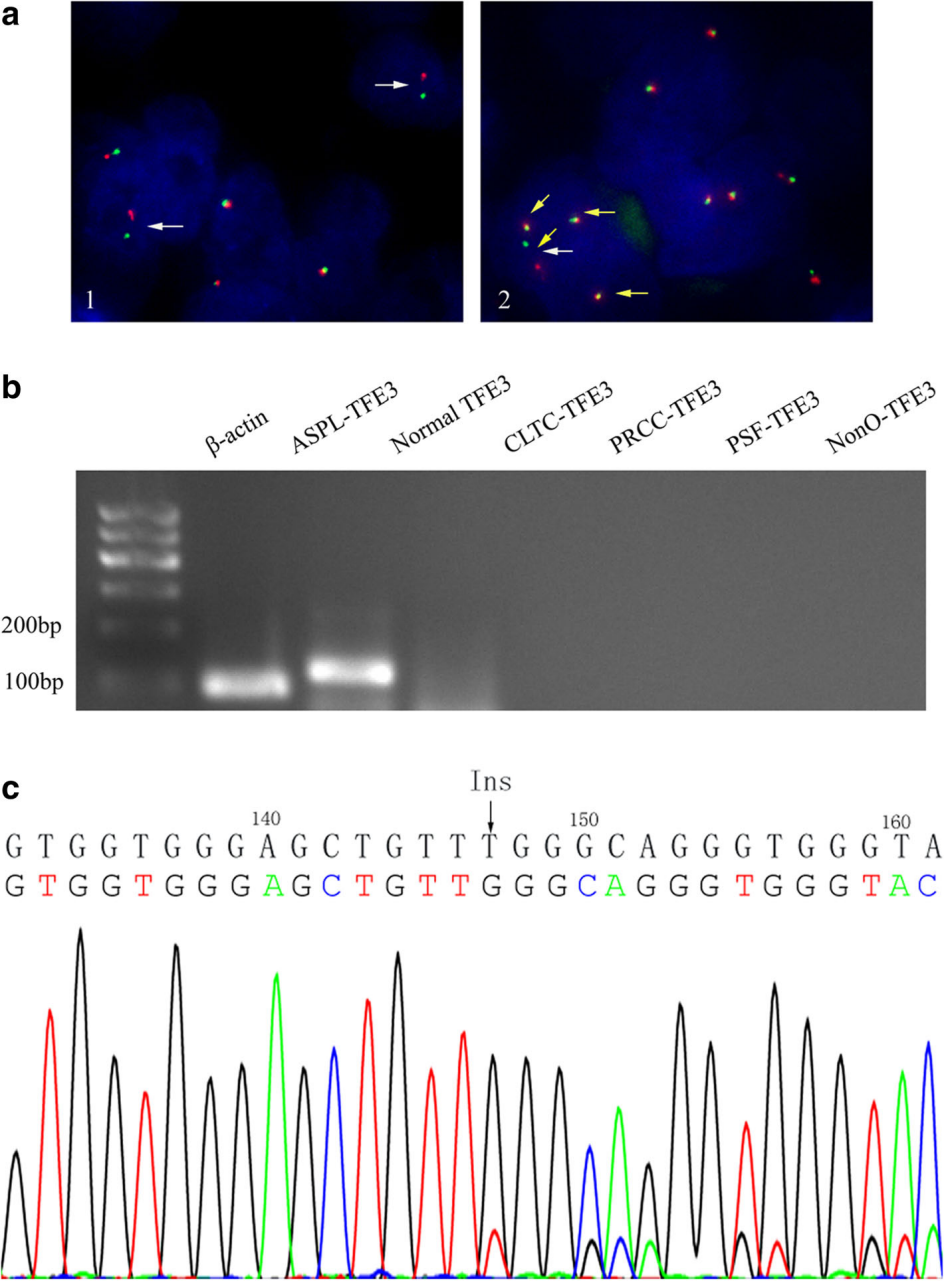
Molecular genetic analyses for the renal tumor. **a** The TFE3 break-apart probe assay identified split signals (white arrow) and increased TFE3 copy numbers (yellow arrow, 4 signals in one nucleus including one split signal). **b** Reverse transcription polymerase chain reaction detected an ASPSCR1-TFE3 fusion gene product. **c** Sequence analysis of SMARCB1 gene (exon 1–9) demonstrated c.147InsT in exon 4, causing a frameshift alteration

To determine the TFE3 rearrangement status, we performed FISH in paraffin-embedded material using a probe comprising 2 contigs flanking the TFE3 gene on Xp11.2 (ZytoLight SPEC TFE3). For males, one fuse signal reflects an intact TFE3 allele. Positivity was defined based on the separation of red and green signals via more than 2 signal diameters (split signals). We used a cutoff of >10 % of the tumor nuclei with any pattern of break-apart signals. In this case, approximately 15 % of the tumor cells showed split signals, and a copy number gain of TFE3 gene was also observed in some tumor cells with or without translocation (3 to 5 signals per nucleus) (Fig. 3a). RNA was extracted from the formalin-fixed and paraffin-embedded tumor samples using a FFPE DNA/RNA Kit (AmoyDx, Guangzhou, China), and subsequently reverse transcription-polymerase chain reaction (RT-PCR) was performed using the PrimeScript™ Reagent kit (Takara, Dalian, Japan). The primers used to detect the gene fusion products of all translocation types (ASPSCR1-TFE3, PRCC-TFE3, PSF-TFE3, CLTC-TFE3 and Nono-TFE3) are listed in Table 1. A band for the ASPSCR1-TFE3 fusion product is demonstrated in Fig. 3b. Thus, an ASPSCR1-TFE3 translocation RCC was considered.

**Table 1:** Primers used to detect the gene fusion product of Xp11.2 translocation RCC

Name	Primer (5' → 3')
β-actin(F)	ATCACCATTGGCAATGAGCG
β-actin(R)	TTGAAGGTAGTTTCGTGGAT
PSF-TFE3(F)	TGGTGGTGGCATAGGTTATG
PSF-TFE3(R)	CGTTTGATGTTGGGCAGCTC
NonO(p54nrb)-TFE3(F)	GAGAAACTAGACACAGCAAC
NonO(p54nrb)-TFE3(R)	CTTTCTTCTGCCGTTCCTTC
PRCC-TFE3(F)	CCAAGCCAAAGAAGAGGA
PRCC-TFE3(R)	AGTGTGGTGGACAGGTACTG
CLTC-TFE3(F)	AGTCGCGTTGTTGGAAAGTATTGTG
CLTC-TFE3(R)	CAAAAGGGCCTTTGCCTCGGTC
ASPSCR1-TFE3(F)	AAAGAAGTCCAAGTCGGGCCA
ASPSCR1-TFE3(R)	CGTTTGATGTTGGGCAGCTCA
Normal TFE3(exon3) (F)	CCCGCAAGTGCCCAGCCACTG
Normal TFE3(exon4) (R)	CAGTTCCTTGATCCTGTCG

In addition to the TFE3 rearrangement, the tumor cells showed the complete loss of SMARCB1 expression. Subsequent DNA sequencing to determine the genetic alteration of *SMARCB1* (exon 1–9) revealed a frameshift mutation in exon 4 of *SMARCB1* (Fig. 3c).

Taken together, a diagnosis of Xp11.2 translocation RCC was preferred. Considering the loss of SMARCB1 expression observed herein, this case presents a unique Xp11.2 translocation RCC with *SMARCB1* inactivation.

This patient died 6 months after the surgery.

## Discussion

Xp11 translocation RCC commonly demonstrates clear cells with papillary, cystic, tubular, and organoid architecture, and psammoma bodies are common in the lesions. Occasionally pleomorphic giant cells and sarcomatoid differentiation are observed. The diagnosis of Xp11 translocation RCC depends on the detection of an Xp11 translocation using a TFE3 break-apart FISH assay performed on paraffin-embedded tissue [1]. Strong nuclear TFE3 immunoreactivity, demonstrating the TFE3 fusion protein resulting from an Xp11 translocation, is helpful for the diagnosis. However, the TFE3 IHC assay is technically challenging, reflecting suboptimal fixation and detection methods, and occasionally TFE3 amplification could serve as additional underlying genomic alterations for TFE3 overexpression [1, 8]. In the present case, the tumor cells with organoid architecture separated by fibrovascular network showed strong TFE3 nuclear staining, a split signal of TFE3 genes, and ASPSCR1-TFE3 fusion PCR products, suggesting Xp11.2 translocation RCC. An increasing number of TFE3 rearrangement-associated tumors, which might morphologically overlap with RCC, including TFE3 rearrangement-associated perivascular epithelioid cell tumors (PEComas) and melanotic Xp11.2 translocation cancer [9, 10], have recently been reported. These tumors can be excluded by immunohistochemistry, as PEComas and melanotic Xp11.2 translocation cancer are negative for renal cell markers (CD10, PAX2 and PAX8) and positive for MelanA and HMB45.

Although a higher percentage of Xp11.2 translocation RCC has been observed in children, adult Xp11.2 translocation RCC is overall more common, reflecting the increased population of adult RCC patients. In adults, the occurrence of Xp11.2 translocation RCC has been correlated with chemotherapy, as approximately 15 % of patients had a history of chemotherapy exposure [1]. The patient described herein had chronic renal failure and received 7 years of hemodialysis. The outcome data for the clinical behavior of adult Xp11.2 translocation RCC remains limited, although adult patients might present a worse prognosis and have a mean survival of up to 2 years when presenting metastases, in contrast to a favorable short-term prognosis and mean survival of 6.3 years in pediatric patients [1]. For adult Xp11.2 translocation RCC, ASPSCR1-TFE3 RCC is more likely to present at an advanced stage compared with PRCC-TFE3 RCC. In addition to TFE3 rearrangement, Macher-Goeppinger S et al. provided evidence that increased TFE3 expression resulting from gene amplification or epigenetic alterations was associated with unfavorable clinicopathological features, such as higher grade, the presence of metastatic disease and advanced tumor stage [8]. This case clearly correlated the presence of metastatic disease with the microscopic appearance of TFE3 overexpression resulted from TFE3 rearrangement and copy number gain. The optimal therapy for the Xp11.2 translocation RCC remains unknown, as clinical studies in a large population of patients remain absent. The current treatment for Xp11.2 RCC generally follows the guidelines of conventional RCC. Therapies targeting vascular endothelial growth factor receptor, C-met or mTOR in Xp11.2 RCC are not yet clear, and additional clinical studies are needed [1, 11].

In addition to cells rich in eosinophilic or clear cytoplasm and arranged in an organoid pattern, some of the tumor cells showed a non-cohesive rhabdoid feature. Accumulating data suggest that tumors with rhabdoid features contain a clinicopathological spectrum of neoplasms with a highly variable rhabdoid cell component, with or without the loss of nuclear SMARCB1 expression. Tumors lacking the biallelic inactivation of the *SMARCB1* gene may develop rhabdoid morphologies. In the kidneys, the morphological rhabdoid differentiation of the primary tumor is typically associated with clear cell RCC and occasionally observed in papillary and chromophobe RCC, collecting duct carcinoma and acquired cystic disease associated RCC, representing a dedifferentiated status that indicates severe cytological anaplasia, a higher stage and a more aggressive biological behavior [1]. SMARCB1-deficient neoplasms might exhibit variable rhabdoid cell components. SMARCB1 deficiency was first observed in renal malignant rhabdoid tumors and childhood CNS AT/RTs. Subsequently the loss of nuclear SMARCB1 expression has been identified in renal medullary carcinoma, epithelioid sarcoma, a subset of collecting duct carcinoma and epithelioid malignant peripheral nerve sheath tumor (MPNST) and rare rhabdoid variants of carcinoma from digestive system and sinonasal tract [2–4, 12–14]. Apparently loss of SMARCB1 has been found in diverse tumors, which have not much in common except the loss of SMARCB1. The diagnosis of those tumors is still mainly based on the phenotype and the clinicopathologic features. Abbas Agaimy proposed that SMARCB1 deficient neoplasms were possibly derived from different progenitor cell subsets with different differentiation commitments in the different organs, and thus probably were genetic subtypes of tumors with certain differentiation [15]. Therefore, a diagnosis of Xp11.2 translocation RCC with SMARCB1 inactivation is proposed in this double hit tumor concerning the histopathological features and TFE3 rearrangement. Such diagnosis doesn’t exclude the possibility that the INI1 loss is the primary event in this tumor.

Most tumors showing the loss of SMARCB1 expression are associated with aggressive biology and poor clinical outcomes. Although the functional role of *SMARCB1* is not completely understood, the loss of SMARCB1 may cause cell cycle progression, and thus this gene might be involved in tumorigenesis via the dysregulation of cell cycle relevant molecules, including p16, Aurora A and cyclin D1, or the induction of DNA repair defects and genomic instability [16]. Concerning the absence of SMARCB1 in different types of tumors, such as epithelioid sarcomas and renal medullary carcinomas, the loss of SMARCB1 expression may be a biological event of uncertain prognostic significance. It is difficult to draw conclusions on the correlation between the loss of SMARCB1 expression and prognosis in the absence of larger studies. A recent finding on targeted therapy revealed that drugs inhibiting cyclin D1 and/or CDKs, such as fenretinide and flavopiridol, are effective in inhibiting rhabdoid tumor growth, correlated with the down-modulation of cyclin D1 [17].

## Conclusion

In summary, both Xp11.2-associated translocation and the loss of SMARCB1 expression in renal cell carcinoma are rare and have not previously been reported. High-grade Xp11.2 RCC with *SMARCB1* inactivation is an extraordinary case, prompting the necessity to elucidate the clinical prognosis and develop therapeutic strategies.

## Abbreviations

AT/RT: Atypical teratoid/rhabdoid tumor
CNS: Central nervous system
FISH: Florescent in situ hybridization
IHC: Immunohistochemistry
MPNST: Epithelioid malignant peripheral nerve sheath tumor
MRI: Magnetic resonance image
MRT: Malignant rhabdoid tumors
PEComas: Perivascular epithelioid cell tumors
RCC: Renal cell carcinoma
RT-PCR: Reverse transcription-polymerase chain reaction
TFE3: Translocation/transcription factor E3
